# (μ_2_-Chlorido)-(μ_2_-pyridine-2-carboxyl­ato-1:2κ*N*,*O*:*O*)-dichlorido(ethanol-κ*O*)bis­[*N*-hy­droxy-1-(pyridin-2-yl)methan­imine-κ^2^ *N*,*N*′]dicobalt(II)

**DOI:** 10.1107/S1600536812041736

**Published:** 2012-10-13

**Authors:** Lei Chen

**Affiliations:** a Shandong Provincial Key Laboratory of Soil Conservation and Environmental Protection, Business School, Linyi University, Linyi 276005, China

## Abstract

The dinuclear title compound, [Co_2_Cl_3_(C_6_H_4_NO_2_)(C_6_H_6_N_2_O)_2_(C_2_H_5_OH)], contains two six-coordinate Co^II^ atoms with different octa­hedral coordination environments. One Co^II^ atom is coordinated by two N atoms from one pyridine-2-carbaldehyde oxime ligand, by one terminal and one bridging Cl^−^ ion, by one O atom from an ethanol mol­ecule, and by one O atom from a bridging pyridine-2-carboxyl­ate (picolinate) anion. The second Co^II^ atom is coordinated by two N atoms from another pyridine-2-carbaldehyde oxime ligand, one N and one O atom from the bridging picolinate anion, and by one terminal Cl^−^ and one bridging Cl^−^ anion. The structure displays intra­molecular O—H⋯O and O—H⋯Cl hydrogen bonds. Weak C—H⋯Cl hydrogen-bonding inter­actions connect the mol­ecules into a three-dimensional network.

## Related literature

For examples of Co^II^ complexes with pyridine-2-carbaldehyde oxime ligands, see: Stamatatos *et al.* (2005*a* ▶,*b* ▶, 2009 ▶); Ross *et al.* (2001 ▶). For the isostructural Ni^II^ analogue, see: Zheng *et al.* (2011 ▶).
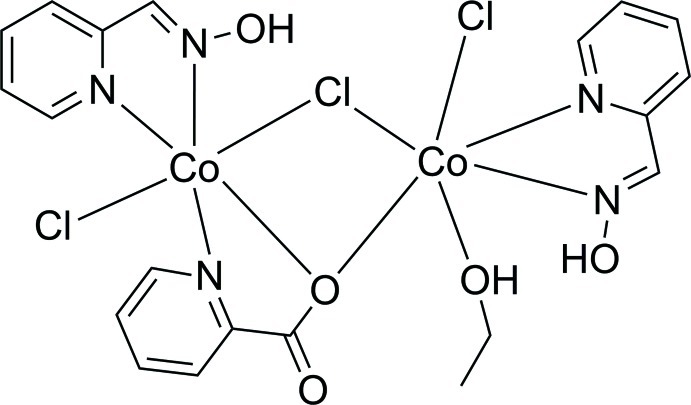


## Experimental

### 

#### Crystal data

[Co_2_Cl_3_(C_6_H_4_NO_2_)(C_6_H_6_N_2_O)_2_(C_2_H_6_O)]
*M* *_r_* = 636.64
Monoclinic, 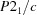

*a* = 8.7443 (17) Å
*b* = 18.144 (4) Å
*c* = 16.643 (3) Å
β = 99.23 (3)°
*V* = 2606.4 (9) Å^3^
*Z* = 4
Mo *K*α radiation
μ = 1.62 mm^−1^
*T* = 293 K
0.30 × 0.25 × 0.21 mm

#### Data collection

Bruker APEXII CCD diffractometer
Absorption correction: multi-scan (*SADABS*; Bruker, 2005 ▶) *T*_min_ = 0.642, *T*_max_ = 0.727
17261 measured reflections
4520 independent reflections
3778 reflections with *I* > 2σ(*I*)
*R*_int_ = 0.026

#### Refinement

*R*[*F* ^2^ > 2σ(*F* ^2^)] = 0.036
*wR*(*F* ^2^) = 0.165
*S* = 1.18
4520 reflections
319 parameters
14 restraints
H-atom parameters constrained
Δρ_max_ = 1.09 e Å^−3^
Δρ_min_ = −1.31 e Å^−3^

### 

Data collection: *APEX2* (Bruker, 2005 ▶); cell refinement: *SAINT* (Bruker, 2005 ▶); data reduction: *SAINT*; program(s) used to solve structure: *SHELXS97* (Sheldrick, 2008 ▶); program(s) used to refine structure: *SHELXL97* (Sheldrick, 2008 ▶); molecular graphics: *SHELXTL* (Sheldrick, 2008 ▶) and *DIAMOND* (Brandenburg, 1999 ▶); software used to prepare material for publication: *SHELXTL*.

## Supplementary Material

Crystal structure: contains datablock(s) I, global. DOI: 10.1107/S1600536812041736/wm2687sup1.cif

Structure factors: contains datablock(s) I. DOI: 10.1107/S1600536812041736/wm2687Isup2.hkl

Additional supplementary materials:  crystallographic information; 3D view; checkCIF report

## Figures and Tables

**Table 1:** Selected bond lengths (Å)

Co1—O3	2.087 (2)
Co1—O5	2.110 (3)
Co1—N1	2.111 (3)
Co1—N2	2.118 (4)
Co1—Cl5	2.3648 (11)
Co1—Cl3	2.4781 (12)
O3—Co2	2.111 (3)
Co2—N3	2.118 (3)
Co2—N5	2.117 (3)
Co2—N4	2.133 (3)
Co2—Cl4	2.3948 (10)
Co2—Cl3	2.4603 (10)

**Table 2:** Hydrogen-bond geometry (Å, °)

*D*—H⋯*A*	*D*—H	H⋯*A*	*D*⋯*A*	*D*—H⋯*A*
O2—H2*A*⋯Cl5	0.82	2.29	3.103 (3)	172
O1—H1⋯O4	0.82	1.83	2.615 (4)	160
O5—H5*A*⋯Cl4	0.85	2.32	3.147 (3)	164
C12—H12⋯Cl4^i^	0.93	2.80	3.684 (4)	160
C10—H10⋯Cl5^ii^	0.93	2.82	3.684 (5)	156
C14—H14⋯Cl4^iii^	0.93	2.74	3.556 (4)	147
C2—H2⋯Cl4^iv^	0.93	2.81	3.654 (5)	152
C17—H17⋯Cl5^v^	0.93	2.80	3.491 (4)	132

Symmetry codes: (i) 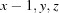; (ii) 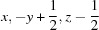; (iii) 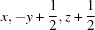; (iv) 
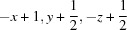
; (v) 
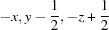
.

**Fig. 1.**
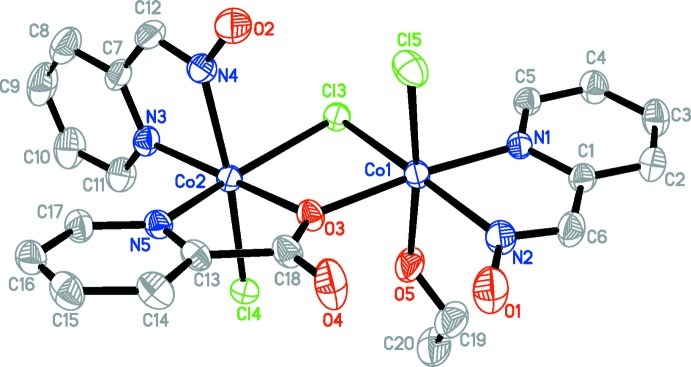
The molecular structure of the title compound, with atom labels and 30% probability displacement ellipsoids.

**Fig. 2.**
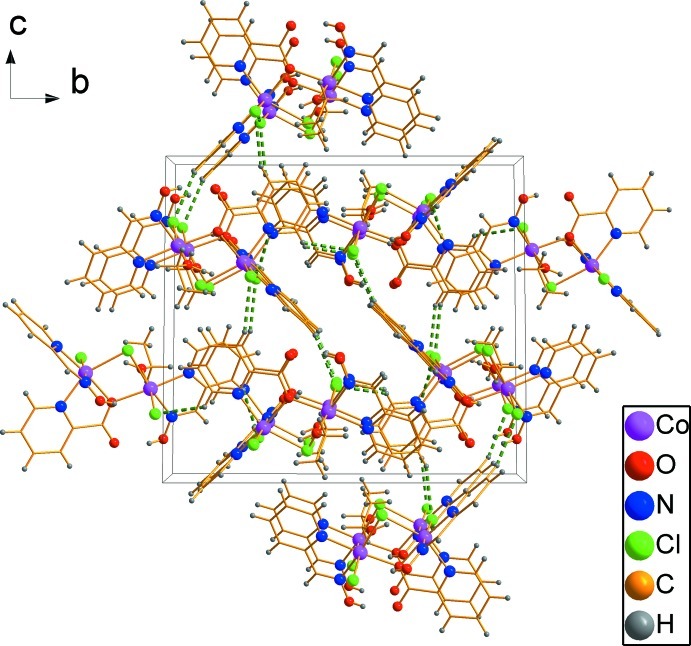
The packing within the structure of compound (I) showing intermolecular C—H···Cl hydrogen bonding interactions.

## supplementary crystallographic information

### Comment

Pyridine-2-carbaldehyde oxime is a frequently used ligand in synthesis of metal complexes. A large number of cobalt complexes based on pyridine-2-carbaldehyde oxime have been reported, such as mononuclear Co complexes (Stamatatos *et al.*, 2005*a*), mixed-valence complexes with trinuclear Co_3_ clusters (Stamatatos *et al.*, 2009), mixed-valence cobalt(III/II/III) complexes with a linear arrangement (Stamatatos *et al.*, 2005*a*), mixed-valence 12-metallacrown-4 complexes (Stamatatos *et al.*, 2005*b*) and some heterodinuclear complexes (Ross *et al.*, 2001). Some of these complexes exhibit interesting magnetic properties (Ross *et al.*, 2001; Stamatatos *et al.*, 2005*b*, 2009). We are interested in the coordination chemistry of cobalt in combination with pyridine-2-carbaldehyde oxime and carboxylic acids. Here, we report a new mixed-bridged binuclear cobalt(II) complex, [Co_2_Cl_3_(C_6_H_4_NO_2_)(C_6_H_6_N_2_O)_2_(C_2_H_5_OH)], (I).

Compound (I) is isostructural with its Ni(II) analogue (Zheng *et al.*, 2011). There are two six-coordinate Co(II) atoms with different coordination environments present in the structure (Fig. 1). The coordination sphere around each Co(II) atom is distorted octahedral. The two Co(II) cations are bridged through one Cl^-^ ion and one carboxylic oxygen atom (O3) from the picolinate anion. The coordination sphere around Co1 is completed by two N atoms from one pyridine-2-carbaldehyde oxime ligand, by one terminal Cl^-^ ion and by one O atom from an ethanol molecule. For Co2 the coordination sphere contains also two N atoms from another pyridine-2-carbaldehyde oxime ligand, one N from the bridging picolinate anion, and one terminal Cl^-^ anion. The Co···Co distance in the dinuclear species is 3.4153 (9) Å.

Intramolecular O—H···O and O—H···Cl hydrogen bonds consolidate the conformation of the complex whereas weak C—H···Cl hydrogen bonding interactions connect the molecules into a three-dimensional network. (Table 2; Fig. 2).

### Experimental

A mixture of CoCl_2_.6H_2_O (0.0476 g, 0.20 mmol), pyridine-2-carbaldehyde oxime (0.0246 g, 0.20 mmol), pyridine-2-carboxylic acid (0.0250 g, 0.20 mmol) and ethanol (4 ml) was sealed into a 10 ml sample bottle reactor and heated at 393 K for 3 d under autogenous pressure, and then cooled to room temperature. Brown block-shaped crystals of the title compound were isolated, washed with distilled water, and dried in air (yield: 25%).

### Refinement

All H atoms were placed in geometrically idealized positions and constrained to ride on their parent atoms with C—H = 0.93 Å for aromatic H atoms, 0.96 Å for CH_3_ type H atoms and 0.97 Å for CH_2_ type H atoms, respectively. *U*_iso_(H) values were set at 1.5*U*eq(C) for methyl H atoms, and 1.2*U*eq(C) for the rest of the H atoms. H atoms bound to O atoms were found from difference maps and were refined with O—H = 0.85 Å and *U*_iso_(H) = 1.2*U*eq(O) for ethanol, and O—H = 0.82 Å and *U*_iso_(H) = 1.5*U*eq(O) for the oxime H atoms.

### Crystal data

**Table:**

[Co_2_Cl_3_(C_6_H_4_NO_2_)(C_6_H_6_N_2_O)_2_(C_2_H_6_O)]	*F*(000) = 1288
*M**_r_* = 636.64	char
Monoclinic, *P*2_1_/*c*	*D*_x_ = 1.622 Mg m^−^^3^
Hall symbol: -P 2ybc	Mo *K*α radiation, λ = 0.71073 Å
*a* = 8.7443 (17) Å	Cell parameters from 3510 reflections
*b* = 18.144 (4) Å	θ = 2.6–25.3°
*c* = 16.643 (3) Å	µ = 1.62 mm^−^^1^
β = 99.23 (3)°	*T* = 293 K
*V* = 2606.4 (9) Å^3^	Block, brown
*Z* = 4	0.30 × 0.25 × 0.21 mm

### Data collection

**Table:**

Bruker APEXII CCD diffractometer	4520 independent reflections
Radiation source: fine-focus sealed tube	3778 reflections with *I* > 2σ(*I*)
Graphite monochromator	*R*_int_ = 0.026
φ and ω scans	θ_max_ = 25.0°, θ_min_ = 2.2°
Absorption correction: multi-scan (*SADABS*; Bruker, 2005)	*h* = −10→7
*T*_min_ = 0.642, *T*_max_ = 0.727	*k* = −21→17
17261 measured reflections	*l* = −18→19

### Refinement

**Table:**

Refinement on *F*^2^	Primary atom site location: structure-invariant direct methods
Least-squares matrix: full	Secondary atom site location: difference Fourier map
*R*[*F*^2^ > 2σ(*F*^2^)] = 0.036	Hydrogen site location: inferred from neighbouring sites
*wR*(*F*^2^) = 0.165	H-atom parameters constrained
*S* = 1.18	*w* = 1/[σ^2^(*F*_o_^2^) + (0.1*P*)^2^] where *P* = (*F*_o_^2^ + 2*F*_c_^2^)/3
4520 reflections	(Δ/σ)_max_ = 0.035
319 parameters	Δρ_max_ = 1.09 e Å^−^^3^
14 restraints	Δρ_min_ = −1.31 e Å^−^^3^

### Special details

**Table:**

Geometry. All e.s.d.'s (except the e.s.d. in the dihedral angle between two l.s. planes) are estimated using the full covariance matrix. The cell e.s.d.'s are taken into account individually in the estimation of e.s.d.'s in distances, angles and torsion angles; correlations between e.s.d.'s in cell parameters are only used when they are defined by crystal symmetry. An approximate (isotropic) treatment of cell e.s.d.'s is used for estimating e.s.d.'s involving l.s. planes.
Refinement. Refinement of *F*^2^ against ALL reflections. The weighted *R*-factor *wR* and goodness of fit *S* are based on *F*^2^, conventional *R*-factors *R* are based on *F*, with *F* set to zero for negative *F*^2^. The threshold expression of *F*^2^ > σ(*F*^2^) is used only for calculating *R*-factors(gt) *etc*. and is not relevant to the choice of reflections for refinement. *R*-factors based on *F*^2^ are statistically about twice as large as those based on *F*, and *R*- factors based on ALL data will be even larger.

### Fractional atomic coordinates and isotropic or equivalent isotropic displacement parameters (Å^2^)

**Table:**

	*x*	*y*	*z*	*U*_iso_*/*U*_eq_	
Co1	0.24774 (6)	0.45656 (2)	0.21872 (3)	0.0402 (2)	
O3	0.2615 (3)	0.34808 (12)	0.26096 (15)	0.0431 (5)	
Co2	0.14638 (5)	0.27859 (2)	0.16890 (3)	0.0375 (2)	
N1	0.2554 (3)	0.56337 (14)	0.16917 (18)	0.0390 (7)	
Cl3	0.08768 (10)	0.39748 (4)	0.09972 (5)	0.0428 (3)	
N5	0.1902 (3)	0.20948 (15)	0.27256 (18)	0.0405 (7)	
Cl4	0.38894 (10)	0.25504 (5)	0.12436 (5)	0.0446 (3)	
N2	0.4077 (4)	0.51273 (17)	0.3068 (2)	0.0532 (8)	
Cl5	0.04241 (13)	0.47676 (5)	0.29287 (7)	0.0599 (3)	
C13	0.2646 (4)	0.24129 (18)	0.3398 (2)	0.0422 (8)	
C5	0.1754 (4)	0.58864 (19)	0.1008 (2)	0.0452 (8)	
H5	0.1081	0.5569	0.0686	0.054*	
O5	0.4324 (4)	0.42495 (16)	0.1587 (2)	0.0755 (9)	
H5A	0.4181	0.3816	0.1395	0.091*	
N3	0.0182 (4)	0.20307 (16)	0.0874 (2)	0.0474 (7)	
C17	0.1477 (5)	0.13908 (19)	0.2775 (3)	0.0499 (9)	
H17	0.0967	0.1159	0.2309	0.060*	
N4	−0.0840 (4)	0.28758 (17)	0.1948 (2)	0.0508 (8)	
C6	0.4351 (5)	0.5793 (2)	0.2900 (3)	0.0539 (10)	
H6	0.5061	0.6073	0.3248	0.065*	
C1	0.3537 (4)	0.61002 (19)	0.2159 (2)	0.0436 (8)	
C2	0.3710 (5)	0.6820 (2)	0.1930 (3)	0.0533 (10)	
H2	0.4399	0.7130	0.2253	0.064*	
C18	0.3098 (5)	0.32080 (19)	0.3299 (2)	0.0473 (7)	
O4	0.3859 (5)	0.35214 (16)	0.3880 (2)	0.0806 (10)	
O2	−0.1396 (4)	0.33138 (18)	0.2505 (2)	0.0702 (9)	
H2A	−0.0851	0.3682	0.2592	0.105*	
C14	0.2986 (5)	0.2054 (2)	0.4128 (2)	0.0534 (10)	
H14	0.3518	0.2291	0.4585	0.064*	
C11	0.0707 (6)	0.1604 (2)	0.0337 (3)	0.0619 (11)	
H11	0.1747	0.1640	0.0285	0.074*	
O1	0.4862 (5)	0.48710 (18)	0.3784 (2)	0.0836 (11)	
H1	0.4460	0.4488	0.3908	0.125*	
C15	0.2517 (5)	0.1328 (2)	0.4166 (3)	0.0560 (11)	
H15	0.2716	0.1069	0.4655	0.067*	
C7	−0.1329 (5)	0.1977 (2)	0.0940 (3)	0.0527 (10)	
C16	0.1765 (5)	0.0998 (2)	0.3486 (3)	0.0546 (10)	
H16	0.1448	0.0510	0.3502	0.065*	
C3	0.2871 (6)	0.7074 (2)	0.1233 (3)	0.0618 (12)	
H3	0.2966	0.7563	0.1080	0.074*	
C4	0.1879 (5)	0.6610 (2)	0.0751 (3)	0.0572 (10)	
H4	0.1304	0.6776	0.0266	0.069*	
C19	0.5751 (6)	0.4495 (3)	0.1537 (4)	0.0872 (12)	
H19A	0.6444	0.4338	0.2021	0.105*	
H19B	0.5732	0.5029	0.1539	0.105*	
C12	−0.1848 (5)	0.2464 (2)	0.1537 (3)	0.0571 (11)	
H12	−0.2878	0.2473	0.1613	0.068*	
C8	−0.2318 (6)	0.1499 (3)	0.0475 (3)	0.0752 (14)	
H8	−0.3356	0.1474	0.0535	0.090*	
C10	−0.0215 (7)	0.1106 (3)	−0.0154 (3)	0.0793 (15)	
H10	0.0197	0.0812	−0.0524	0.095*	
C20	0.6395 (7)	0.4247 (3)	0.0818 (4)	0.0869 (17)	
H20A	0.6291	0.3722	0.0766	0.130*	
H20B	0.7471	0.4379	0.0879	0.130*	
H20C	0.5842	0.4479	0.0339	0.130*	
C9	−0.1751 (7)	0.1058 (3)	−0.0078 (4)	0.0886 (17)	
H9	−0.2401	0.0730	−0.0399	0.106*	

### Atomic displacement parameters (Å^2^)

**Table:**

	*U*^11^	*U*^22^	*U*^33^	*U*^12^	*U*^13^	*U*^23^
Co1	0.0441 (3)	0.0344 (3)	0.0410 (3)	−0.00033 (19)	0.0033 (3)	−0.00025 (17)
O3	0.0526 (13)	0.0361 (11)	0.0379 (12)	0.0020 (10)	−0.0012 (11)	0.0034 (9)
Co2	0.0386 (3)	0.0373 (3)	0.0360 (3)	−0.00170 (18)	0.0039 (3)	0.00143 (17)
N1	0.0405 (16)	0.0351 (13)	0.0410 (17)	−0.0009 (12)	0.0053 (14)	−0.0003 (12)
Cl3	0.0450 (5)	0.0427 (5)	0.0389 (5)	−0.0019 (4)	0.0013 (4)	0.0037 (3)
N5	0.0423 (16)	0.0403 (14)	0.0388 (17)	0.0020 (12)	0.0059 (14)	0.0061 (12)
Cl4	0.0408 (5)	0.0464 (5)	0.0470 (6)	0.0052 (4)	0.0082 (4)	−0.0003 (4)
N2	0.060 (2)	0.0443 (17)	0.049 (2)	−0.0008 (15)	−0.0098 (17)	0.0022 (14)
Cl5	0.0740 (7)	0.0520 (5)	0.0591 (7)	0.0109 (5)	0.0272 (6)	0.0026 (4)
C13	0.050 (2)	0.0398 (16)	0.038 (2)	0.0077 (16)	0.0088 (18)	0.0047 (14)
C5	0.046 (2)	0.0465 (18)	0.041 (2)	−0.0026 (16)	0.0009 (18)	−0.0007 (15)
O5	0.0564 (16)	0.0550 (15)	0.124 (3)	−0.0128 (13)	0.0415 (18)	−0.0243 (16)
N3	0.0502 (19)	0.0450 (15)	0.0437 (18)	−0.0074 (14)	−0.0024 (16)	0.0040 (13)
C17	0.054 (2)	0.0426 (19)	0.053 (2)	−0.0017 (17)	0.007 (2)	0.0066 (16)
N4	0.0475 (19)	0.0470 (16)	0.061 (2)	0.0025 (15)	0.0181 (18)	0.0093 (15)
C6	0.061 (2)	0.0441 (19)	0.052 (2)	−0.0105 (18)	−0.004 (2)	−0.0081 (17)
C1	0.044 (2)	0.0435 (18)	0.043 (2)	−0.0015 (15)	0.0060 (17)	−0.0062 (15)
C2	0.063 (2)	0.0456 (19)	0.051 (2)	−0.0122 (18)	0.007 (2)	−0.0038 (17)
C18	0.0573 (18)	0.0397 (15)	0.0412 (16)	0.0055 (14)	−0.0031 (16)	0.0011 (13)
O4	0.128 (3)	0.0466 (15)	0.0528 (19)	−0.0108 (18)	−0.029 (2)	0.0031 (13)
O2	0.0638 (19)	0.0657 (19)	0.088 (2)	0.0002 (15)	0.0337 (19)	−0.0059 (16)
C14	0.067 (3)	0.055 (2)	0.039 (2)	0.0097 (19)	0.009 (2)	0.0035 (16)
C11	0.071 (3)	0.059 (2)	0.055 (3)	−0.009 (2)	0.008 (2)	−0.0079 (19)
O1	0.108 (3)	0.0595 (19)	0.066 (2)	−0.0138 (18)	−0.037 (2)	0.0093 (15)
C15	0.070 (3)	0.053 (2)	0.049 (2)	0.008 (2)	0.020 (2)	0.0179 (18)
C7	0.047 (2)	0.053 (2)	0.055 (3)	−0.0141 (18)	−0.002 (2)	0.0151 (18)
C16	0.056 (2)	0.049 (2)	0.060 (3)	0.0027 (18)	0.016 (2)	0.0178 (18)
C3	0.082 (3)	0.0406 (19)	0.062 (3)	−0.008 (2)	0.009 (3)	0.0075 (19)
C4	0.068 (3)	0.048 (2)	0.052 (2)	−0.001 (2)	−0.001 (2)	0.0091 (17)
C19	0.062 (2)	0.077 (2)	0.130 (3)	−0.0157 (18)	0.038 (2)	−0.028 (2)
C12	0.039 (2)	0.060 (2)	0.073 (3)	−0.0074 (19)	0.011 (2)	0.013 (2)
C8	0.064 (3)	0.078 (3)	0.078 (3)	−0.027 (3)	−0.007 (3)	0.006 (3)
C10	0.104 (4)	0.068 (3)	0.063 (3)	−0.021 (3)	0.005 (3)	−0.020 (2)
C20	0.082 (4)	0.077 (3)	0.112 (5)	−0.012 (3)	0.048 (4)	−0.013 (3)
C9	0.099 (4)	0.077 (3)	0.079 (4)	−0.040 (3)	−0.018 (3)	−0.004 (3)

### Geometric parameters (Å, º)

**Table:**

Co1—O3	2.087 (2)	C6—H6	0.9300
Co1—O5	2.110 (3)	C1—C2	1.376 (5)
Co1—N1	2.111 (3)	C2—C3	1.350 (6)
Co1—N2	2.118 (4)	C2—H2	0.9300
Co1—Cl5	2.3648 (11)	C18—O4	1.223 (5)
Co1—Cl3	2.4781 (12)	O2—H2A	0.8200
O3—C18	1.258 (4)	C14—C15	1.384 (5)
O3—Co2	2.111 (3)	C14—H14	0.9300
Co2—N3	2.118 (3)	C11—C10	1.388 (7)
Co2—N5	2.117 (3)	C11—H11	0.9300
Co2—N4	2.133 (3)	O1—H1	0.8200
Co2—Cl4	2.3948 (10)	C15—C16	1.354 (6)
Co2—Cl3	2.4603 (10)	C15—H15	0.9300
N1—C5	1.319 (5)	C7—C8	1.372 (6)
N1—C1	1.358 (5)	C7—C12	1.455 (6)
N5—C17	1.337 (4)	C16—H16	0.9300
N5—C13	1.333 (5)	C3—C4	1.371 (6)
N2—C6	1.271 (5)	C3—H3	0.9300
N2—O1	1.359 (5)	C4—H4	0.9300
C13—C14	1.369 (5)	C19—C20	1.473 (7)
C13—C18	1.512 (5)	C19—H19A	0.9700
C5—C4	1.391 (5)	C19—H19B	0.9700
C5—H5	0.9300	C12—H12	0.9300
O5—C19	1.339 (5)	C8—C9	1.371 (8)
O5—H5A	0.8499	C8—H8	0.9300
N3—C11	1.319 (5)	C10—C9	1.371 (8)
N3—C7	1.347 (5)	C10—H10	0.9300
C17—C16	1.370 (6)	C20—H20A	0.9600
C17—H17	0.9300	C20—H20B	0.9600
N4—C12	1.269 (6)	C20—H20C	0.9600
N4—O2	1.368 (4)	C9—H9	0.9300
C6—C1	1.435 (6)		
			
O3—Co1—O5	84.04 (11)	O2—N4—Co2	129.1 (3)
O3—Co1—N1	173.45 (10)	N2—C6—C1	118.2 (4)
O5—Co1—N1	89.42 (12)	N2—C6—H6	120.9
O3—Co1—N2	102.98 (11)	C1—C6—H6	120.9
O5—Co1—N2	89.32 (14)	N1—C1—C2	121.4 (4)
N1—Co1—N2	76.69 (12)	N1—C1—C6	115.5 (3)
O3—Co1—Cl5	88.74 (7)	C2—C1—C6	123.1 (4)
O5—Co1—Cl5	172.77 (9)	C3—C2—C1	119.5 (4)
N1—Co1—Cl5	97.81 (8)	C3—C2—H2	120.2
N2—Co1—Cl5	92.09 (10)	C1—C2—H2	120.2
O3—Co1—Cl3	81.72 (7)	O4—C18—O3	126.8 (3)
O5—Co1—Cl3	83.16 (10)	O4—C18—C13	118.4 (3)
N1—Co1—Cl3	97.71 (9)	O3—C18—C13	114.8 (3)
N2—Co1—Cl3	170.71 (10)	N4—O2—H2A	109.5
Cl5—Co1—Cl3	96.05 (4)	C13—C14—C15	118.0 (4)
C18—O3—Co1	132.1 (2)	C13—C14—H14	121.0
C18—O3—Co2	118.5 (2)	C15—C14—H14	121.0
Co1—O3—Co2	108.89 (11)	N3—C11—C10	123.3 (4)
O3—Co2—N3	173.23 (10)	N3—C11—H11	118.3
O3—Co2—N5	76.08 (11)	C10—C11—H11	118.3
N3—Co2—N5	98.43 (12)	N2—O1—H1	109.5
O3—Co2—N4	99.41 (12)	C16—C15—C14	119.3 (4)
N3—Co2—N4	76.05 (13)	C16—C15—H15	120.3
N5—Co2—N4	86.20 (12)	C14—C15—H15	120.3
O3—Co2—Cl4	89.33 (7)	N3—C7—C8	122.5 (4)
N3—Co2—Cl4	95.19 (9)	N3—C7—C12	115.4 (4)
N5—Co2—Cl4	95.35 (8)	C8—C7—C12	122.1 (4)
N4—Co2—Cl4	171.24 (10)	C15—C16—C17	119.3 (4)
O3—Co2—Cl3	81.68 (7)	C15—C16—H16	120.3
N3—Co2—Cl3	102.62 (9)	C17—C16—H16	120.3
N5—Co2—Cl3	153.82 (8)	C2—C3—C4	119.9 (4)
N4—Co2—Cl3	83.95 (9)	C2—C3—H3	120.0
Cl4—Co2—Cl3	98.06 (3)	C4—C3—H3	120.0
C5—N1—C1	118.5 (3)	C3—C4—C5	118.2 (4)
C5—N1—Co1	127.6 (2)	C3—C4—H4	120.9
C1—N1—Co1	113.9 (2)	C5—C4—H4	120.9
Co2—Cl3—Co1	87.51 (4)	O5—C19—C20	115.5 (5)
C17—N5—C13	117.7 (3)	O5—C19—H19A	108.4
C17—N5—Co2	126.9 (3)	C20—C19—H19A	108.4
C13—N5—Co2	115.3 (2)	O5—C19—H19B	108.4
C6—N2—O1	115.6 (4)	C20—C19—H19B	108.4
C6—N2—Co1	115.6 (3)	H19A—C19—H19B	107.5
O1—N2—Co1	128.9 (2)	N4—C12—C7	117.4 (4)
N5—C13—C14	123.2 (3)	N4—C12—H12	121.3
N5—C13—C18	115.0 (3)	C7—C12—H12	121.3
C14—C13—C18	121.9 (4)	C9—C8—C7	119.0 (5)
N1—C5—C4	122.5 (4)	C9—C8—H8	120.5
N1—C5—H5	118.8	C7—C8—H8	120.5
C4—C5—H5	118.8	C9—C10—C11	118.1 (5)
C19—O5—Co1	136.9 (3)	C9—C10—H10	120.9
C19—O5—H5A	111.5	C11—C10—H10	120.9
Co1—O5—H5A	110.5	C19—C20—H20A	109.5
C11—N3—C7	117.7 (4)	C19—C20—H20B	109.5
C11—N3—Co2	127.4 (3)	H20A—C20—H20B	109.5
C7—N3—Co2	114.8 (3)	C19—C20—H20C	109.5
N5—C17—C16	122.4 (4)	H20A—C20—H20C	109.5
N5—C17—H17	118.8	H20B—C20—H20C	109.5
C16—C17—H17	118.8	C10—C9—C8	119.3 (5)
C12—N4—O2	114.6 (3)	C10—C9—H9	120.3
C12—N4—Co2	116.3 (3)	C8—C9—H9	120.3
			
O5—Co1—O3—C18	107.7 (3)	N5—Co2—N3—C11	−95.8 (4)
N2—Co1—O3—C18	19.8 (3)	N4—Co2—N3—C11	−179.7 (4)
Cl5—Co1—O3—C18	−72.1 (3)	Cl4—Co2—N3—C11	0.4 (3)
Cl3—Co1—O3—C18	−168.4 (3)	Cl3—Co2—N3—C11	99.9 (3)
O5—Co1—O3—Co2	−80.18 (14)	N5—Co2—N3—C7	82.3 (3)
N2—Co1—O3—Co2	−168.10 (12)	N4—Co2—N3—C7	−1.6 (3)
Cl5—Co1—O3—Co2	100.04 (10)	Cl4—Co2—N3—C7	178.5 (3)
Cl3—Co1—O3—Co2	3.74 (8)	Cl3—Co2—N3—C7	−82.0 (3)
C18—O3—Co2—N5	3.5 (3)	C13—N5—C17—C16	0.8 (5)
Co1—O3—Co2—N5	−169.85 (13)	Co2—N5—C17—C16	−177.4 (3)
C18—O3—Co2—N4	87.2 (3)	O3—Co2—N4—C12	−174.2 (3)
Co1—O3—Co2—N4	−86.18 (13)	N3—Co2—N4—C12	0.7 (3)
C18—O3—Co2—Cl4	−92.2 (3)	N5—Co2—N4—C12	−99.0 (3)
Co1—O3—Co2—Cl4	94.47 (9)	Cl3—Co2—N4—C12	105.3 (3)
C18—O3—Co2—Cl3	169.6 (3)	O3—Co2—N4—O2	5.2 (3)
Co1—O3—Co2—Cl3	−3.77 (8)	N3—Co2—N4—O2	−179.9 (3)
O5—Co1—N1—C5	92.3 (3)	N5—Co2—N4—O2	80.4 (3)
N2—Co1—N1—C5	−178.2 (3)	Cl3—Co2—N4—O2	−75.3 (3)
Cl5—Co1—N1—C5	−87.9 (3)	O1—N2—C6—C1	−178.9 (3)
Cl3—Co1—N1—C5	9.3 (3)	Co1—N2—C6—C1	1.8 (5)
O5—Co1—N1—C1	−88.9 (2)	C5—N1—C1—C2	−0.3 (5)
N2—Co1—N1—C1	0.5 (2)	Co1—N1—C1—C2	−179.1 (3)
Cl5—Co1—N1—C1	90.8 (2)	C5—N1—C1—C6	179.0 (3)
Cl3—Co1—N1—C1	−171.9 (2)	Co1—N1—C1—C6	0.2 (4)
O3—Co2—Cl3—Co1	3.01 (7)	N2—C6—C1—N1	−1.3 (5)
N3—Co2—Cl3—Co1	177.68 (9)	N2—C6—C1—C2	178.0 (4)
N5—Co2—Cl3—Co1	35.0 (2)	N1—C1—C2—C3	1.0 (6)
N4—Co2—Cl3—Co1	103.47 (11)	C6—C1—C2—C3	−178.3 (4)
Cl4—Co2—Cl3—Co1	−85.12 (4)	Co1—O3—C18—O4	−12.9 (6)
O3—Co1—Cl3—Co2	−3.04 (7)	Co2—O3—C18—O4	175.5 (4)
O5—Co1—Cl3—Co2	81.89 (10)	Co1—O3—C18—C13	166.0 (2)
N1—Co1—Cl3—Co2	170.37 (8)	Co2—O3—C18—C13	−5.5 (4)
Cl5—Co1—Cl3—Co2	−90.87 (4)	N5—C13—C18—O4	−176.1 (4)
O3—Co2—N5—C17	177.6 (3)	C14—C13—C18—O4	3.9 (6)
N3—Co2—N5—C17	1.7 (3)	N5—C13—C18—O3	4.9 (5)
N4—Co2—N5—C17	76.9 (3)	C14—C13—C18—O3	−175.2 (3)
Cl4—Co2—N5—C17	−94.4 (3)	N5—C13—C14—C15	−0.7 (6)
Cl3—Co2—N5—C17	145.0 (2)	C18—C13—C14—C15	179.4 (3)
O3—Co2—N5—C13	−0.5 (2)	C7—N3—C11—C10	−0.4 (7)
N3—Co2—N5—C13	−176.5 (2)	Co2—N3—C11—C10	177.7 (4)
N4—Co2—N5—C13	−101.2 (3)	C13—C14—C15—C16	0.9 (6)
Cl4—Co2—N5—C13	87.4 (2)	C11—N3—C7—C8	0.2 (6)
Cl3—Co2—N5—C13	−33.2 (4)	Co2—N3—C7—C8	−178.1 (3)
O3—Co1—N2—C6	172.0 (3)	C11—N3—C7—C12	−179.5 (4)
O5—Co1—N2—C6	88.3 (3)	Co2—N3—C7—C12	2.2 (4)
N1—Co1—N2—C6	−1.2 (3)	C14—C15—C16—C17	−0.4 (6)
Cl5—Co1—N2—C6	−98.8 (3)	N5—C17—C16—C15	−0.5 (6)
O3—Co1—N2—O1	−7.2 (4)	C1—C2—C3—C4	−1.3 (6)
O5—Co1—N2—O1	−90.9 (4)	C2—C3—C4—C5	0.9 (6)
N1—Co1—N2—O1	179.5 (4)	N1—C5—C4—C3	−0.2 (6)
Cl5—Co1—N2—O1	82.0 (4)	Co1—O5—C19—C20	−158.8 (4)
C17—N5—C13—C14	−0.2 (5)	O2—N4—C12—C7	−179.2 (3)
Co2—N5—C13—C14	178.2 (3)	Co2—N4—C12—C7	0.3 (5)
C17—N5—C13—C18	179.8 (3)	N3—C7—C12—N4	−1.7 (6)
Co2—N5—C13—C18	−1.9 (4)	C8—C7—C12—N4	178.7 (4)
C1—N1—C5—C4	−0.1 (5)	N3—C7—C8—C9	0.0 (7)
Co1—N1—C5—C4	178.6 (3)	C12—C7—C8—C9	179.6 (5)
O3—Co1—O5—C19	−126.6 (6)	N3—C11—C10—C9	0.4 (8)
N1—Co1—O5—C19	53.2 (6)	C11—C10—C9—C8	−0.2 (8)
N2—Co1—O5—C19	−23.5 (6)	C7—C8—C9—C10	0.0 (8)
Cl3—Co1—O5—C19	151.0 (6)		

### Hydrogen-bond geometry (Å, º)

**Table:**

*D*—H···*A*	*D*—H	H···*A*	*D*···*A*	*D*—H···*A*
O2—H2*A*···Cl5	0.82	2.29	3.103 (3)	172
O1—H1···O4	0.82	1.83	2.615 (4)	160
O5—H5*A*···Cl4	0.85	2.32	3.147 (3)	164
C12—H12···Cl4^i^	0.93	2.80	3.684 (4)	160
C10—H10···Cl5^ii^	0.93	2.82	3.684 (5)	156
C14—H14···Cl4^iii^	0.93	2.74	3.556 (4)	147
C2—H2···Cl4^iv^	0.93	2.81	3.654 (5)	152
C17—H17···Cl5^v^	0.93	2.80	3.491 (4)	132

Symmetry codes: (i) *x*−1, *y*, *z*; (ii) *x*, −*y*+1/2, *z*−1/2; (iii) *x*, −*y*+1/2, *z*+1/2; (iv) −*x*+1, *y*+1/2, −*z*+1/2; (v) −*x*, *y*−1/2, −*z*+1/2.
